# Presidential Symposium: Disruption to Transformation of Communities of Care: Key Issues and Opportunities in Health Sciences

**DOI:** 10.1093/geroni/igab046.1123

**Published:** 2021-12-17

**Authors:** Christine Mueller, Kirsten Corazzini, Jennifer Wolff

## Abstract

Consistent with the theme of the conference this year, this symposium explores the impact of the pandemic and our commitment to redress structural racism and health inequities on health of older adults, and our collective capacity to transform and innovate through our gerontological health sciences lens. Each presenter will focus on one sector of care: (1) the health system and healthcare workforce; (2) older persons; and (3) families and care partners. The first presenter provides a systems-level perspective on key disruptors in healthcare systems for care of older adults and the workforce, and emerging innovations to address increasingly transparent care inequities. Emerging research implications as a result of these disruptions will be highlighted. The second presenter highlights how the predominant features of the pandemic in older adults, loneliness and isolation, are co-occurring with significant resilience and innovation, and the resultant potential to create a paradigm shift in how we design and advance communities of care. The third presenter provides the perspective of family members and care partners of older adults during the time of the pandemic, focusing on disruptions that have informed changes going forward, innovations that have emerged, and implications for research to be addressed. Our discussant situates the presentations within the larger context of ‘the new normal’, with a particular focus on considering the global, inter-connected context of communities of care, a commitment to reducing inequities for older adults, and implications for health sciences education and policy.

